# Acute, but not constitutive, loss of endothelial β3-integrin inhibits tumour growth and angiogenesis

**DOI:** 10.1186/1753-6561-7-S2-O7

**Published:** 2013-04-04

**Authors:** Veronica Steri, Tim Ellison, Katherine Weilbaecher, Kairbaan Hodivala-Dilke, Stephen D Robinson

**Affiliations:** 1 University of East Anglia, School of Biological Sciences, Norwich, UK; 2 Washington University, St Louis, USA; 3 Queen Mary’s University London, Barts Cancer Institute, London, UK

## Background

Angiogenesis, the formation of new vessels from pre-existing ones, is essential for tumour growth and metastasis. Endothelial cells play a central role in this process: they drive blood vessel formation in response to signals from the local environment, by a mechanism that is integrin-dependent. We are particularly interested in understanding what role αvβ3-integrin plays in governing tumour angiogenesis.

αvβ3-integrin seemingly poses an ideal anti-angiogenic target. Its expression is vastly up-regulated in neo-angiogenic vessels, while its expression in quiescent vasculature is minimal. However, anti-angiogenic therapy targeting αvβ3-integrin has proven somewhat disappointing. In part this likely relates to the fact that αvβ3-integrin is not expressed solely by endothelial cells, but across a wide range of cell types that each contribute to angiogenesis. The aim of the research we present here is to elucidate the role of αvβ3-integrin in tumour growth and angiogenesis as it is expressed specifically by endothelial cells.

## Methods

We have crossed β3-integrin floxed animals to two Cre transgenic models to delete β3-integrin specifically in endothelial cells. In Pdgfb.Creert2 transgenic mice, β3-integrin is deleted in a tamoxifen-inducible fashion in neo-angiogenic endothelial cells, while in Tie1.Cre transgenic mice β3-integrin is constitutively deleted in endothelial cells. In these animals, we have studied angiogenesis via the *ex vivo* aortic ring model, and in subcutaneoulsy grown B16F0 and CMT19T allograft tumours.

## Results

In β3-floxed/Pdgfb.Creert2 positive mice treated with tamoxifen, allograft tumours grow significantly smaller when compared to their growth in Cre negative littermate controls. This correlates with decreased microvascular density observed in Cre-postive compared to Cre-negative tumour sections. In contrast, constitutive deletion of endothelial β3-integrin via Tie1.Cre leads to enhanced tumour angiogenesis. These findings are re-capitulated in the aortic ring model. VEGF-induced microvessel sprouting is inhibited in β3-floxed/Pdgfb.Creert2 positive mice compared to Cre-negative littermates. In marked contrast, sprouting is enhanced in β3-floxed/Tie1.Cre positive mice compared to β3-floxed/Tie1.Cre negative mice.

## Conclusions

These findings strengthen the argument that endothelially-expressed β3-integrin remains a valid target of anti-angiogenic tumour therapy. However, taken together, these data suggest that the timing and length of exposure to β3-integrin endothelial genetic inhibition impacts on the angiogenic response. These data highlight the need to enhance our understanding of the molecular basis of angiogenesis in order to develop improved therapeutic treatments.

## Financial support

University of East Anglia and the BigC Norfolk Cancer Charity.

